# Survey of Foreign Body Aspiration in Airways and Lungs

**DOI:** 10.5539/gjhs.v6n7p130

**Published:** 2014-09-18

**Authors:** R. Samarei

**Affiliations:** 1 Head & Neck Surgeon, Urmia University of Medical Science, Urmia, Iran

**Keywords:** foreign body aspiration, rigid bronchoscopy

## Abstract

**Introduction:**

Foreign body aspiration is a very serious problem and the diversity of clinical protests in each geographic region has its own characteristics and common problems of childhood that is an important cause of mortality and morbidity. No area is separate from this problem and conducting this research is due to achieve basic information regarding foreign body aspiration.

**Materials and Methods:**

This was performed as descriptive - cross sectional study on 200 cases that has been hospitalized in Imam Khomeini Hospital, Urmia due to foreign body aspiration problem from 2009 to 2011. And all cases of foreign body aspiration records extracted and analyzed using SPSS 16 software.

**Results:**

Foreign body aspiration under 4 years was 57% and was more common in males than females; approximately 74% of patients were hospitalized in the first 10 days and 13% of patients did not remember the initial incident that led to the aspiration. Cough and shortness of breath and reduced lung sounds and wheezing were common symptoms. Chest radiographic findings are not specific and can be normal of a high percentage. The most common aspirated foreign body was food especially sunflower seeds. Right bronchus with 55% of cases was more common than the left bronchus and all patients were treated with rigid bronchoscopy, 24% of patients had complications, 15% had hospitalized with pneumonia. Totally, 75% of patients were urban residents.

**Discussion:**

We need to understand all the aspects related to foreign body aspiration and education to the community, to recognize symptoms and type of foreign body in terms of geographical area and to create a strong clinical suspicion in physicians and awareness of its prevalence that by reducing the incidence and early detecting and treatment can reduce the mortality and morbidity and prevent additional expenses.

## 1. Introduction

Foreign body aspiration into the tracheobronchial tract is usually more than the rate to be diagnosed. Most patients with foreign body aspiration are children under 4 years, and most cases of deaths caused by foreign body aspiration are also in this age. These children often put things in their mouths and often stay away from even the sharpest vision care. Also at this age due to lack of proper neuromuscular coordination of the throat, foreign body aspiration into the airways are more likely. Because the right main bronchus is placed straighter than the left bronchus due to chip, so foreign bodies are more likely to enter the right lung but due to this straightness, possibility of exiting foreign body from right bronchus following cough is more than left bronchus and therefore in various articles the prevalence of bronchial foreign bodies has evaluated almost equal in both bronchus (Michael & Paparella, 2012). Usually, foreign body aspiration manifests as a stable and persistent cough without phlegm (although in some cases it may be due to damage of the airways associated with hemoptysis). If the foreign body aspiration is big or associated with laryngospasm it can cause respiratory distress and cyanosis (Richard, 2011). Foreign body is rarely big enough to cause obstruction and choking. In most cases foreign bodies are non-blocking and just cause stridor and audible breaths. Sometimes because the air is entered into the lungs during inhalation, but during exhalation due to mucosal edema of the airways air duct diameter is reduced and the air does not exit and this phenomenon is caused Obstructive Emphysema. Sometimes due to the lack of airflow during inhalation and exhalation, the air is absorbed and eventually in the blocked area, atelectasis is caused (Kruk-Zagajewska, 1998). In case of emphysema, signs of CXR will be as hyperinflation and mediastina shift to the other side and in case of atelectasis, signs of CXR will be as increased density of the affected area and the shift of the mediastinum to the affected area. When a child refers with a chronic cough and sputum, foreign body airway should be considered. The first diagnostic step is patient’s history, whether there is previous record of foreign body aspiration and choking or not, which in case of presence will inform us for the possibility of a foreign body aspiration (Michael & Paparella, 2012)

When the obstruction is incomplete during inhalation and exhalation, there would not be sufficient X-ray evidence in favor of a foreign body, unless the foreign body is opaque and visible in the CXR. Performance of a neck and chest X-ray will help us isolate the foreign body (Richard, 2011). Performing CXR in decubitus will help in the detection of foreign bodies. In general, the diagnostic approach to foreign body is that if a foreign body is suspected, according to the case history, but due to lack of opaqueness of foreign body CXR be normal, bronchoscopy should be performed, if the foreign body is not found in bronchoscopy but is still suspected, CT scan of the lung is requested. Flexible bronchoscopy is also used for diagnostic purposes. Removal of foreign body in the lungs of children to achieve better results should be performed by Rigid Bronchoscopy, because this type of bronchoscopy allows proper ventilation of patient during bronchoscopy. Due to the narrow subglottic in children, bronchoscopy that is easily traversable from that area is selected (Richard, 2011). In a study that was conducted at the Department of ENT, Medical College Karnataka-Davanger India, foreign body aspiration was a very serious problem with a variety of clinical protests that needs clinical suspicion for diagnosis. Surgical emphysema in the neck and chest often complicate the tracheostomy and penetrating injuries to the neck caused by sharp objects that damage the airways and gastrointestinal tract. The results of the study showed that cervical emphysema following foreign body aspiration into the airways is quite rare. However, in this study, four cases of surgical emphysema have been reported following foreign body aspiration into the tracheobronchial tree (Shiva Kumar, 2004).

In a research that was conducted in 2002 in Brazil, foreign body aspiration in children is a common phenomenon and is one of the major causes of morbidity and mortality. High clinical suspicion is needed to diagnose and the clinical and radiographic studies have low sensitivity in diagnosing the problem. This is especially the case in children younger than 3 years that are much more common in boys. Aspirated material usually includes food, especially grains such as peanuts, although the type of aspirated food is different due to regional habits. The study also showed that the right bronchus is the most common place where the foreign body is found. Radiographic findings are non-specific; however, the presence of unilateral obstructive emphysema with atelectasis is an important diagnostic sign if the history is oriented toward the foreign body aspiration. Rigid bronchoscopy is recommended and delay in the removal of foreign body may lead to severe bronchial schematic. In developing countries, these phenomena may be much higher due to the lack of awareness and lack of resources that can lead to delayed diagnosis and treatment. The results of this study showed that educating patients under the care and health care education and giving awareness to people have important role in decreasing the incidence of foreign body aspiration (Shiva, 2004).The most common foreign bodies were including bone, kidney bean, beans, fruit cores, flakes, food, chips and pieces of iron. Removal of 4 cases of foreign bodies occurred spontaneously. 96% of foreign bodies were removed by rigid bronchoscopy. Flexible bronchofiberoscopy was performed for diagnostic purposes or removing foreign bodies in the peripheral parts of bronchial tree. 9% of patients were referred to conduct trachea surgery (after bronchoscopy) (Kruk-Zagajewska, 1998). A study that is conducted at the ENT Department of Medical University in Bulgaria showed the association between chronic pneumonia and foreign body airways based on 480 bronchoscopy studies that have been conducted over a period of 15 years. The rate of misdiagnosis in foreign body lower airways is assessed by about 12.5%. Misdiagnosis of bilateral lung foreign body and also foreign body in the left lung is more (even if the percentage is too low). The study also shows that in many cases foreign body aspiration in children is wrongly diagnosed as bronchopneumonia and asthma (Tsolou, 1999). Chronic cough can be indicative of an alarming condition that should be diagnosed as soon as possible and without delay, like cystic fibrosis, asthma, bronchiectasis, and bronchial foreign body. The purpose of this study is to investigate the frequency of symptoms and CXR findings of foreign body airways.

## 2. Materials and Methods

This was performed as descriptive-cross sectional study on 200 cases that has been hospitalized in Imam Khomeini Hospital, Urmia due to foreign body aspiration problem from 2009 to 2011 and the data was collected by referring to the hospital archives and studying the cases related to foreign body on ENT. Collecting data was performed based on all records of pulmonary foreign body aspiration that were hospitalized since 2011, in Imam Khomeini Hospital and only incomplete records were deleted. And all cases of foreign body aspiration were extracted from files and above information has been analyzed using SPSS 16 software (Dalfard et al., 2012).

## 3. Results

According to the results of the descriptive statistics that was performed on 200 patients, the following results were obtained:

The age survey of patients in 5 age groups of less than 1 year (10%), 1 to 2 years (47%) and 2 to 4 years (23%) and 4 to 10 (12%) and more than 10 years were (8%) (Table 1).

**Table 1:** Distribution of absolute and relative frequency of cases by age

Age group					Frequency

More than 10 years	4 to 10 years	2 to 4 years	1 to 2 years	Less than 1 year	
16	24	46	94	20	Absolute
8%	12%	23%	47%	10%	Relative

In this study, of 200 patients, 60% of the patients were male and 40% were women, and in the study in terms of location, 75% were living in urban centers and 25% of the patients were from rural areas. Patients were evaluated in terms of two groups, those who recall the initial incident, and those who do not remember that were included 87% and 13%, respectively. The group that remembers the incident was evaluated in 7 time groups, including: Less than 1 day, 1 to 10 days, 10 to 20 days, 20 days to 1 year, 1 month to 2 months, 2 months to 1 year, and over 1 year that were accounted for 35%, 39%, 2%, 4%, 4%, and 2%, 1% respectively (Table 2).

**Table 2:** Distribution of relative and absolute frequency of study subjects according to the reference after aspiration (incident recalls)

Reference period after aspiration							Frequency

More than 1 year	2 months- 1 year	1 month- 2 months	20 days- 1 month	10 to 20 days	1 to 10 days	Less than 1 day	
2	4	8	8	4	78	70	Absolute
1	2	4	4	2	39	35	Relative

The group does not remember the original incident was under review at 1-7 days, 7 days to 1 month, 1 month to 1 year, and more than 1 year that was 2%, 1%, 7% and 3% respectively (Table 3).

**Table 3:** Distribution of absolute frequency of study subjects according to the reference after the onset of clinical signs (do not remember the initial event)

Reference period after the onset of clinical signs				Frequency

More than 1 year	1 month to 1 year	7 days to 1 month	1-7 days	
6	14	2	4	Absolute

The patient had symptoms and signs that were found on examination, were evaluated to assess the symptoms: Cough in75% of patients, dyspnea in 53% of patients, bruising in 7.5%, wheezing in 2%, non-bloody sputum in 9%, hemoptysis in 1%, chronic cough in 13%, interrupted breathing in 5%, vomiting in 8%, and halitosis in 2% have mentioned (Table 4).

**Table 4:** Distribution of relative and absolute frequency of study subjects according to type of clinical signs

Clinical signs	Frequency
Cough	150
Dyspnea	106
Bruising	15
Wheezing	4
Non-bloody sputum	18
Chronic cough	26
Hemoptysis	2
Interrupted breathing	10
Vomiting	16
Halitosis	4

The signs: Decreased lung sounds in 35% of patients, wheezing in 21%, fever in 15%, rales in 19%, respiratory distress in 17%, stridor in 3%, impaired consciousness in 1%, and respiratory and cardiac arrest with 1% prevalence have been found in the examination (Table 5).

**Table 5:** Distribution of relative and absolute frequency of symptoms of study subjects that were found on examination

Symptoms	Frequency	Frequency percentage
Fever	30	15
Crackles	38	19
Noise reduction	70	35
Wheezing	42	21
Respiratory distress	34	17
Stridor	6	3
Impaired consciousness	2	1
Respiratory and cardiac arrest	2	1

In radiographic examination 37% of patients had normal radiography and in 38% of patients with radiographic alveolar opacities and foreign bodies found in 4% of patients and in 12% of patients with emphysema and 3% of patients pleural effusion and 5% atelectasis and in 1% of patients pneumothorax was observed on chest radiography (Table 6).

**Table 6:** Distribution of absolute and relative frequency of radiologic signs in the subjects

Frequency	Radiologic findings						

	Normal	Alveolar opacity	Foreign body	Emphysema	Pleural effusion	Atelectasis	Pneumothorax
Absolute	74	76	8	24	6	10	2
Relative	37	38	4	12	3	5	1

In the case of foreign body removal, all is done with a rigid bronchoscopy. We had no treatment with flexible bronchoscopy or thoracotomy. The following results were obtained in terms of the foreign body type, sunflower seeds 47%, 13% were uncertain objects, pieces of carrots 3%, and pulses such as beans, peas 6%, pen stub 5%, seed beads 6%, and walnut 3%, (Table 7).

**Table 7:** Distribution of absolute and relative frequency of subjects in terms of foreign body type

Frequency	Type of foreign body						

	Walnut	Seed beads	Pen stub	Pulses (beans, peas)	Pieces of carrots	Unknown	Sunflower seeds
Absolute	6	12	10	12	6	26	98
Relative	3	6	5	6	3	13	49

The establishment of foreign body in right bronchus was 55% and in left bronchus was 30% and 9% in both bronchi and the trachea was 7%. The location of the foreign body in the right bronchus was observed in most cases (Table 8).

**Table 8:** Distribution of relative and absolute frequency based on the location of foreign body in the subjects

Frequency	Location of foreign body			

	Chip	Right and left bronchus simultaneously	Left bronchus	Right bronchus
Absolute	12	18	60	110
Relative	6	9	30	55

In the survey of the effects of foreign body aspiration, 24% of patients had complications related to aspiration and of these 15% had pneumonia, 5% had atelectasis, and 3% had pleural effusion and pneumothorax in 1% of patients (Table 9).

**Table 9:** Distribution of relative and absolute frequency of type of associated complications in these patients

Frequency	Type of associated complications			

	Pneumothorax	Pleural effusion	Atelectasis	Pneumonia
Absolute	2	6	10	30
Relative	1	3	5	15

## 4. Discussion

The research team conducted a study on 200 patients with a diagnosis of FBA (Foreign Bodies Aspiration) that were hospitalized from January 2009 to December 2011 at the Medical Center of Imam Khomeini, and the foreign body was extracted with rigid bronchoscopy. In an age survey in this study the most age spread was under 4 years that consists 80% of patients which the highest number was under 2 years with 57% that was consistent with the literature that have mentioned the most common age under 4 years. In article 3, which was conducted in Poznan, Poland, 68% of patients were under 10 years of age which were included 92% of patients in our study. Perfect nervous - muscular pharynx harmony in children under 4 years is not complete and due to the lack of awareness in this age, they put everything in their mouth. In terms of sex, rate of aspiration was 60% in males that according to a study in Brazil, the rate in males was much more than females perhaps due to the high mobility and the lack of control of boys is justifiable (Shiva Kumar, 2004).

In terms of reference and hospitalization, about 74% of patients referred in first 10 days that from these 35% referred first day and 39% referred from second day to tenth day. According to the study conducted in Poland in 86.5% of patients were hospitalized in first 3 days, which could be due to differences in the level of public awareness (Kruk-Zagajewska, 1998). FBA patients who were hospitalized at the time of admission, 75% had presented with cough and 53% with dyspnea that include the most common symptoms and the most common findings on physical examination were lung volume reduction of 35% and wheezing of 21% that is consistent with the literature discussed in scientific texts include most common signs and symptoms. The most common radiographic findings were alveolar opacity with 38% and normal cases in graph were 37%. Note that the type of foreign body is food and is non-opaque we expect no specific radiographic findings and our findings on radiographs were also nonspecific. According to the literature and study in Poland, graph has mentioned nonspecific that cannot help to a definitive diagnosis (David, 2013). In our study, the most common aspirated foreign body was foods that commonly sunflower seeds were allocated to 49%.

That was consistent with the literature and the study of Polish that have expressed food as the most commonly aspirated foreign bodies. And from food, snacks and nuts were more than the rest and this is expected because snacks and nuts are in exposure of children for amusement (Dutau, 2001). Given that eating habits plays a role in the type of aspirated foreign body based on our region who are planting sunflower seeds in our area and when they eat it tear to pieces and due to poor neurodevelopmental - muscular pharynx, which can be justified why sunflower seeds are common. Right bronchus because of its more direct route, is a common site for the entry of foreign bodies that in our study and the literature cited the most common area and in our article with 55% right main bronchus was the most common. In our study the removal of all foreign bodies was by the rigid bronchoscope that is the best way to cure and of the benefits is possible ventilation during bronchoscopy. The incidence of patients who were evaluated, 24% were complicated that pneumonia was the most common complication with 15%, and atelectasis with 5% was in the next place. In terms of residence in review, the majority of patients, 75% of patients were urban and 25% were of village.

## 5. Recommendations

In all of the results that were derived from our study, is the same or close to the literature and the results of research in other countries and slight differences in the results can be attributed to the difference between public awareness and access to facilities of equipped centers, therefore, it is recommended:

1)- The best and least cost way, is raising public awareness for meticulous care of their children and recommendations concerning swallow objects that have been kept away from children and not to use food and nuts for children’s entertainment.
2)- In the diagnosis dimension considering that chest radiography is nonspecific and place of objects are in the main bronchi and trachea with low percentage rigid bronchoscope can be used as the first diagnostic and therapeutic procedure to reduce additional costs and time, to reduce hospital stay and to decrease mortality.
3)- In future studies in this field, radiography can be examined separately in patients who can recall the original incident and those who do not and compare the results and measure the radiation sensitivity.
